# Evaluating the impact of extended dosing intervals on mRNA COVID-19 vaccine effectiveness in adolescents

**DOI:** 10.1186/s12916-024-03597-4

**Published:** 2024-09-12

**Authors:** Tim K. Tsang, Sheena G. Sullivan, Yu Meng, Francisco Tsz Tsun Lai, Min Fan, Xiaotong Huang, Yun Lin, Liping Peng, Chengyao Zhang, Bingyi Yang, Kylie E. C. Ainslie, Benjamin J. Cowling

**Affiliations:** 1 WHO Collaborating Centre for Infectious Disease Epidemiology and Control, School of Public Health, Li Ka Shing, Faculty of Medicine, The University of Hong Kong, 7 Sassoon Road, Pokfulam, Hong Kong Special Administrative Region China; 2 Laboratory of Data Discovery for Health Limited, Hong Kong Science and Technology Park, New Territories, Hong Kong Special Administrative Region, China; 3 School of Clinical Sciences, Monash University, Melbourne, Australia; 4 Department of Epidemiology, University of California, Los Angeles, USA; 5 Centre for Safe Medication Practice and Research, Department of Pharmacology and Pharmacy, Li Ka Shing Faculty of Medicine, The University of Hong Kong, Hong Kong Special Administrative Region, China; 6 Centre for Infectious Disease Control, National Institute for Public Health and Environment (RIVM), Bilthoven, the Netherlands

**Keywords:** COVID-19, MRNA vaccine, SARS-CoV-2, Vaccination, Vaccine effectiveness, Extended dosing interval

## Abstract

**Background:**

Extending the dosing interval of a primary series of mRNA COVID-19 vaccination has been employed to reduce myocarditis risk in adolescents, but previous evaluation of impact on vaccine effectiveness (VE) is limited to risk after second dose.

**Methods:**

We quantified the impact of the dosing interval based on case notifications and vaccination uptake in Hong Kong from January to April 2022, based on calendar-time proportional hazards models and matching approaches.

**Results:**

We estimated that the hazard ratio (HR) and odds ratio (OR) of infections after the second dose for extended (28 days or more) versus regular (21–27 days) dosing intervals ranged from 0.86 to 0.99 from calendar-time proportional hazards models, and from 0.85 to 0.87 from matching approaches, respectively. Adolescents in the extended dosing groups (including those who did not receive a second dose in the study period) had a higher hazard of infection than those with a regular dosing interval during the intra-dose period (HR 1.66; 95% CI 1.07, 2.59; *p* = 0.02) after the first dose.

**Conclusions:**

Implementing an extended dosing interval should consider multiple factors including the degree of myocarditis risk, the degree of protection afforded by each dose, and the extra protection achievable using an extended dosing interval.

**Supplementary Information:**

The online version contains supplementary material available at 10.1186/s12916-024-03597-4.

## Background

For most COVID-19 vaccines, the primary vaccination series consists of two doses, separately administered over weeks or months. The second dose is essential for increasing the immunogenicity and effectiveness of these vaccines [1, 2]. Some countries have extended the recommended interval between the first and second doses of COVID-19 vaccines to more rapidly increase the proportion of the population who have had at least one dose (i.e., dose-sparing) [3, 4] and minimize the risk of myocarditis after vaccination, especially in children and adolescents [5–8].

Extending the dosing interval has been associated with a stronger neutralizing antibody response [9, 10] and higher vaccine effectiveness (VE) [11–13]. Test-negative design studies in Canada have reported a 5–10% absolute increase in VE for adults (aged 18 +) and adolescents (aged 12–17) who received their primary series vaccinations 49 days or more apart (extended dosing interval) compared to those who received their primary series 21–48 days apart (regular dosing interval) [12, 13]. Similarly, a study in the UK reported minor increments in VE (5–10% absolute increase) against hospitalization among individuals who were vaccinated according to an extended versus regular dosing interval (Table S1). In contrast, a nested case–control study in Hong Kong [11] reported an odds ratio (OR) for infection of 0.7 for children and adolescents with a 28 days or more gap between doses (extended dosing interval) compared to 27 days (regular dosing interval), corresponding to increase in VE of 30%.

Clarifying the degree of extra protection gained from extending dosing interval is important for vaccine policy. The discrepancies between the Hong Kong study and other studies may be due to differences in geography or differences in public health policy context. In Hong Kong, the first large community outbreak occurred in January–April 2022, which was dominated by the Omicron variant. In this outbreak, the Hong Kong government adopted a “work from home” policy for government workers which was followed by most of the private sector. People were also self-isolated for self-protection [14, 15]. Despite this, there were more than 1 million reported cases in this outbreak. The decision to extend the dosing interval in Hong Kong occurred in November 2021 prior to this outbreak. Consequently, by the time the epidemics occurred, a majority of adolescents vaccinated under a regular dosing interval would also have experienced a longer time since vaccination. Therefore, it may also be biased due to inappropriate handling of VE waning. It is well established that the effectiveness of these vaccines wanes with time [16, 17]. Failure to handle this waning effect could have artificially inflated the apparent gains from an extended dosing interval because those in the regular interval group would have experienced more time since vaccination and therefore more opportunity for waning, and hence lower VE compared with extended interval group. This problem could be avoided by restricting the comparison group for an extended dosing interval to a time-matched regular-interval comparison group with the same duration of follow-up since second dose. Instead, the Hong Kong study used an adjustment approach that put time since vaccination of second dose as a covariate in the model [11].

A further consideration for studies wishing to examine the protective benefits of extending the primary vaccination series is whether the extended interval between doses may increase the opportunity for infection during the inter-dose period. Most of the studies described in Table S1 only counted the time since dose 2; they did not consider infections that occurred during the inter-dose period and therefore did not assess whether the extended dose group was also at greater risk of infection while waiting for their second dose. Again, a calendar-time-matched comparison group, at equal risk of infection because they experience vaccination at the same stage of the epidemic, could permit estimation of the increased risk of infection among those in the extended dosing interval group, thereby providing a more reliable estimate of the true gains of an extended dose interval.

Here, we conducted a comprehensive analysis on adolescents (aged 12–17 years) who received their primary of mRNA vaccination series in Hong Kong to evaluate the impact of extending dosing intervals. We first estimated protection from an extended versus regular dosing intervals since receipt of the second dose (the relative VE of an extended versus regular dosing intervals). We used various methods, including a calendar-time proportional hazards model and case–control approaches with case-density sampling, to handle waning VE and estimate this protection. We also conducted simulation studies to compare these methods and evaluate their validity. Then, we used calendar-time proportional hazard model to evaluate the increased infection risk during the inter-dose period.

## Methods

We aimed to determine whether primary vaccination with extended dosing intervals provides higher protection than regular intervals for adolescents aged 12–17 receiving mRNA vaccines. Specifically, we examined [1] whether primary vaccination with an extended dosing interval provides higher protection against infections after receiving the second dose compared to primary vaccination with a regular dosing interval and [2] whether the increased risk during the inter-dose period due to an extended dosing interval may counterbalance the additional protection gained from extending the dosing interval. Analysis was restricted to the age group because > 99% of children aged 0–11 receiving mRNA vaccines with the interval between first dose and second dose ≥ 56 days.

### Study population and vaccination eligibility

Hong Kong had a population of 7.4 million people, including 389,400 adolescents aged 12–17 years at the end of 2021 [18]. Adolescents became eligible for COVID-19 vaccination on 4 April 2021 and were able to receive CoronaVac (Sinovac Biotech) and Comirnaty® (BNT162b2, Pfizer-BioNTech) vaccines. Initially, the recommended dosing interval was 21 days. To reduce the myocarditis risk associated with mRNA COVID-19 vaccine, the recommended dosing interval was extended to 84 days from 23 December 2021. The recommended dosing interval was reduced to 56 days from 17 June 2022 [11].

### Data sources

We obtained a COVID-19 database from the Center of Health Protection (CHP) in Hong Kong, which included vaccination records and case details linked by unique identifiers. In Hong Kong, COVID-19 vaccination records and positive test results for SARS-CoV-2 were required to be reported to the CHP from January 2020 to January 2023 [14]. Positive rapid test results were required to be reported to CHP from 26 February 2022 to 29 January 2023. Rapid tests were available to purchase and free kits were distributed by the government. Both datasets contained demographic and relevant medical information, including date of birth, sex, and underlying conditions. Vaccinees were required to reporting underlying conditions before they received vaccination. We excluded adolescents with underlying conditions to avoid potential confounding associated with preferential vaccination for people with underlying conditions.

The vaccination dataset included individuals with any recorded vaccination, detailing the date and type of vaccine for each dose. We assumed that each vaccine dose required 14 days to become effective. Those vaccinated within 14 days of SARS-CoV-2 notification were considered unvaccinated; i.e., observation time commenced 14 days after the second dose. Individuals infected within 14 days after dose 2 vaccination were excluded in the analysis. Adolescents were considered to have received a regular dosing schedule if the interval between their first and second dose was 21–27 days, while an extended interval was assigned if doses were received more than or equal to 28 days apart.

The case dataset included all confirmed COVID-19 cases (PCR or RAT) in Hong Kong, with their notification date, hospitalization outcome, and mortality outcome (all-cause or COVID-related). Cases detected prior to 1 January 2022 were excluded from the analysis because all were infected by ancestral strains in 2020–2021 and the incidence rate was very low (12,631 cases among 7 million residents) [14, 15].

### Observed relative vaccine effectiveness of extended versus regular dosing interval since second dose

Statistical analyses were performed using R version 4.0.5 (R Foundation for Statistical Computing, Vienna, Austria). Individuals infected prior to receipt of their second dose during the Omicron outbreaks were excluded due to potential hybrid immunity [19]. Data were analyzed to estimate the relative hazard of infection among adolescents receiving vaccination according to an extended versus regular dosing schedule. Analyses were restricted to the period 1 January to April 30, 2022, marking the first Omicron wave and prior to the introduction of new circulating Omicron variants in May 2022. During this period, all RAT-confirmed cases required a mandatory confirmatory PCR test. We assumed that each individual could only be infected once during the fifth wave between January and April 2022 [20, 21]. In all analysis, we assumed each vaccine dose required 14 days to be effective.

We employed a Cox proportional hazard model to estimate the hazard ratio (HR) of infection for extended versus regular dosing intervals. To account for the varying infection risk during an epidemic the time-to-event was based on calendar time, with observation time commencing 1 January 2022 and ending on the date of case notification or 30 April 2022. Individuals started to contribute person-time to the analysis 14 days after receiving their second dose and after 1 January 2022 [22]. The exposure was the vaccination dosing interval, dichotomized to regular (21–27 days) and extended (28 + days). In sensitivity analyses, the threshold was varied to 56 days (see below). To account for waning VE, time since vaccination was included as a time-varying term, calculated as the number of days since 14 days post-second-dose. Other variables included were age and sex, which were treated as time-independent variables. Therefore, the equation of the cox model was:

$$h\left(t\right)={h}_{0}\left(t\right)*\text{exp}({\upbeta }_{1}*Z+{\beta }_{2}*sex+{\beta }_{3}*age+{\beta }_{4}*f(X))$$

where $Z$ was the indicator of extended dosing group, $X$ was the days since second dose of vaccination, and $f$ is the function of waning, ${\upbeta }_{1}$, ${\upbeta }_{2}$, ${\upbeta }_{3}$, and ${\upbeta }_{4}$ were the effects associated with extending dosing group, sex, age, and VE waning respectively.

**Fig. 1:**
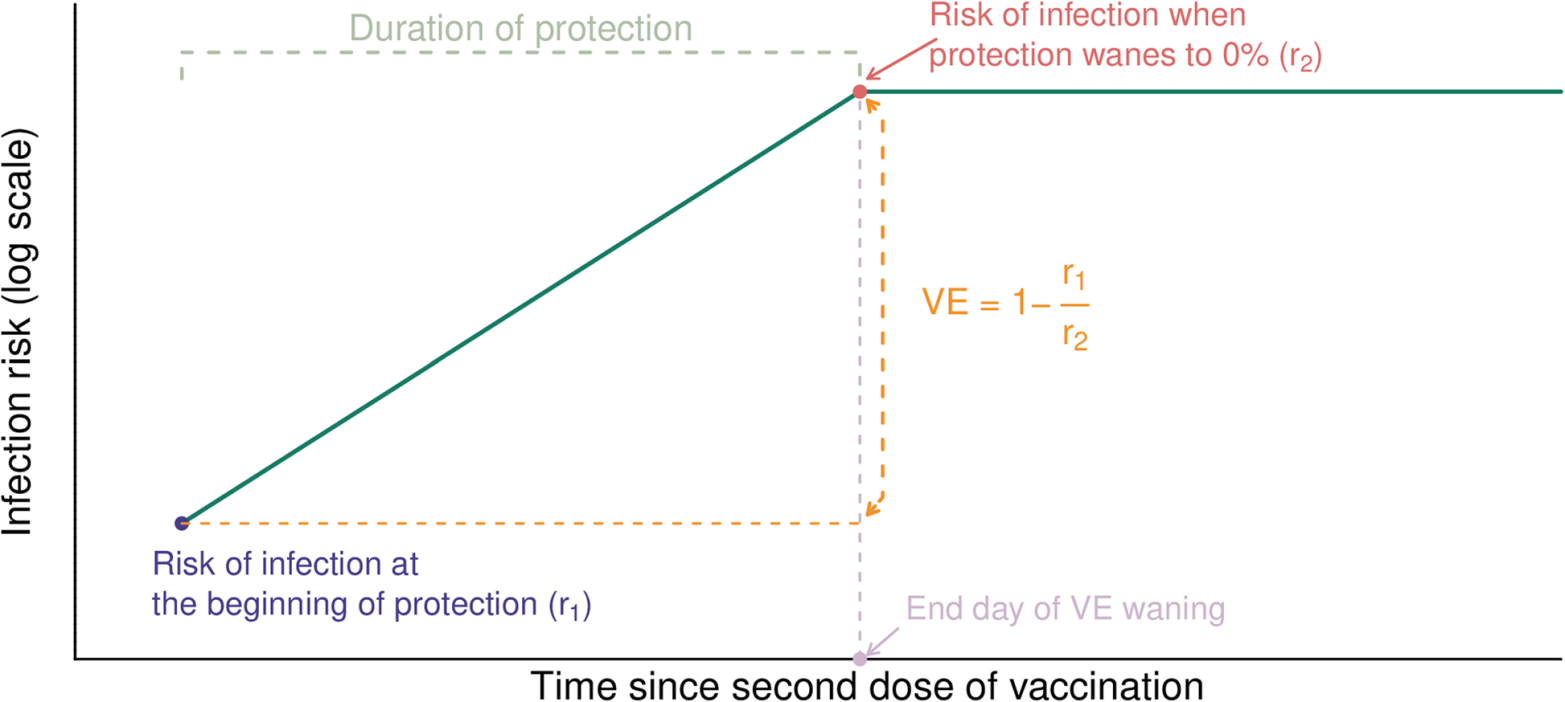
Study flowchart

Unvaccinated adolescents were not considered; therefore, VE estimates of primary series were therefore projected backward to day 0 based on the estimated waning rate. Primary series VE was derived from the relative risk of infection at 14 days after vaccination (r_1_), versus the risk of infection at the end of VE waning (r_2_); i.e., VE = 1 − r_1_/r_2_ (Fig. 1). The risk of infection was assumed to increase over time consistent with waning VE, such that r_1_ < r_2_. We tested the impact on different assumptions on primary series VE and VE waning, or allowed them to be estimated from data. In our main analysis, when the end day of waning was set to *Y* days, the assumed function of waning was *f(X)* = *minimum(X,Y)*, and put into the regression, so that the infection risk increased log-linearly from *r*_*1*_ to *r*_*2*_ in *Y* days, and protection could reach 0% by 90 or 180 days and then stay at zero thereafter. This regression coefficient was estimated, and therefore when it was estimated to be positive, the infection risk was increasing since second dose and hence the primary series VE was positive, and vice versa. When waning was incorporated into the model as a time-varying linear term defined by the number of days since second dose with an assumed function of *f(X)* = *X*. There was no upper bound to the number of days since second dose; i.e., waning was assumed to never end and decline beyond zero to negative values (replicating the assumptions of Lai et al. [11]).

VE was estimated from the model at different assumptions about waning, where protection was assumed to wane to 0% perpetually or within 90 or 180 days, since previous studies suggest that VE wanes to negligible around 90–180 days [23–27]. Waning after 90 days in the regular dosing interval group and 180 days in the extended dosing interval group was also modeled, to test the potential of reducing waning rate from extended dosing intervals. In addition, we explored the effect of waning when VE was set to 40% or 25% [28–31]. We conducted sex-specific analyses and sensitivity analyses. We varied the threshold for the dosing interval to 56 days instead of 28 days and excluded participants with extreme dosing intervals (> 100 days). We conducted sensitivity analyses that fitting the models restricted to 90 or 180 days after vaccination.

We tested a case-density sampling approach, which allows cases to be selected as controls during their period at risk (i.e., prior to infection) in a matching analysis. This approach can accommodate the time-varying infection risk observed during epidemics. Also, this approach can address potential “positivity” [32] may arise because of the non-overlapping periods during which both groups could potentially become infected. The case-to-control ratio was 1:4, matched by age and sex. Specifically, for each case with a known infection date, we randomly chose 4 control individuals who had not been infected by that date and shared the same age and sex as the case. In these simulations, we examined whether the HR could be reliably approximated by the odds ratio (OR). Two conditional logistic regression models were explored. In the first, the data were additionally matched by the date of the second vaccine dose, which more closely resembles density sampling. In the second, days since the second dose was included as an unmatched covariate and incorporated into the model as a time-varying linear term, as used by Lai et al. [11].

### Simulation study for validation of approaches

To assess the impact of approaches or assumptions on primary series VE or duration of protection in estimating the protection of extended versus regular dosing intervals, we developed a simulation model to test different estimation approaches to determine if they could provide unbiased estimates (Supplementary information). This model described infection risk since the first dose, assuming risk was proportional to community case numbers. We then excluded individuals with infections before their second dose, mimicking the construction of the real dataset. We tested the true value of HR = 1 and 0.85, corresponding to no effect and moderate effect of extended versus regular dosing interval.

### Observed relative vaccine effectiveness of extended versus regular dosing interval since first dose

Restricting the comparison of extended versus regular dosing intervals to the infection risk since the second dose ignores the potential increased risk of infection during the inter-dose interval. Therefore, we used the same calendar-time proportional hazard model to evaluate the impact of increased risk of infection during the inter-dose period. In this analysis, individuals infected prior to receipt of their first dose during the Omicron outbreaks were excluded due to potential hybrid immunity [19]. We compared the infection risk for adolescents who received vaccination 21–27 days since first dose (regular dosing interval) versus those were not, including those who receive a second dose 28 days or more after first dose (extended dosing interval), or who did not. Two ranges of intervals were examined: [1] 42–98 days, consistent with the recommendation to separate doses by 84 days from 23 December 2021 and adopted during the study period, and [2] 42–70 days after first dose, corresponding to a 28-day inter-dose interval + 14 days to allow for seroconversion, and a 56 days interval, which was the interval recommended by the Hong Kong government from 17 June 2022.

### Comparison of infection risk since first dose instead of second dose based on simulation studies

Given that almost all adolescents (98%) who received vaccination in 2022 had an extended dosing interval and the Omicron outbreak in Hong Kong also started in January 2022, comparing the risk of infection since first dose among adolescents with regular and extended dosing intervals in the real dataset may not be robust. Therefore, we further used simulations to compare the risk of infection during the inter-dose period for the extended and regular dosing groups. In the simulation, the VEs of primary series were ranged from 0 to 45%, the HR of infection of extended versus regular dosing intervals ranged from 0.6 to 1, and duration of protection were set to 90 days in both vaccination groups. In a sensitivity analysis the duration of protection was extended to 180 days for the extended dosing group.

## Results

### Study participants

**Fig. 2:**
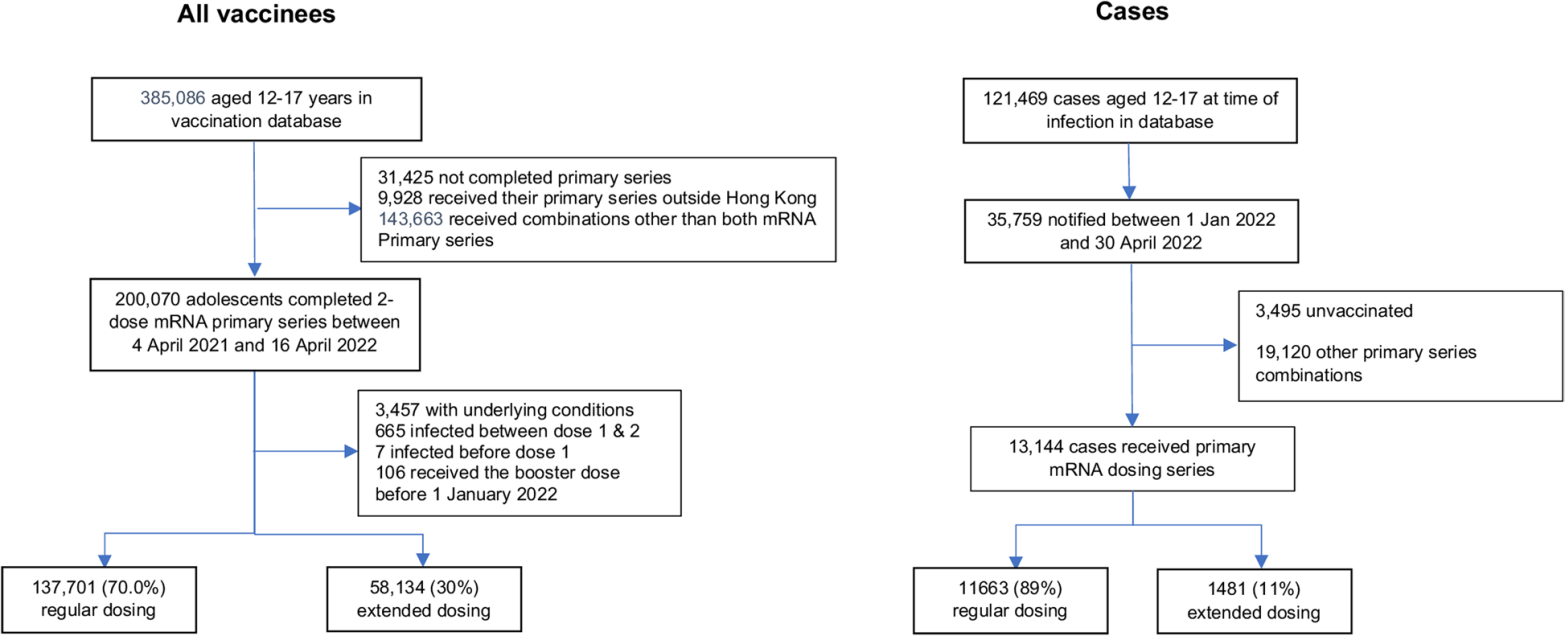
Time series of mRNA-vaccinations, COVID-19 cases, hospitalizations, and deaths among adolescents aged 12–17 years in Hong Kong, March 10, 2021, to April 30, 2022. **A** The time series of first and second doses of mRNA vaccine, categorized by regular dosing interval (< 28 days) or extended dosing intervals (28 days or more). **B** The time series of COVID-19 cases by vaccination status. **C** The number of COVID-19 hospitalizations and deaths

Between 4 April 2021 and 30 April 2022, 385,086 adolescents aged 12–17 years had completed their primary vaccination series (Fig. 2), of whom 200,070 received 2 doses of an mRNA vaccines. Excluded from further analysis were 3457 with at least one underlying condition, 665 participants infected between dose 1 and 2, seven infected prior to dose 1, and 106 who received their booster dose before 1 January 2022 (the start of the Omicron outbreak). Of the remaining 195,835, 137,701 (70%) completed a regular dosing series, while 58,134 (30%) completed an extended series (Fig. 2A). The recommended inter-dose interval was changed on 23 Dec 2021; therefore, the distribution of those completing a regular versus extended dosing series was not consistent over time, and 95% (136,944/144,831) of adolescents vaccinated in 2021 were vaccinated according to a regular dosing interval (21–27 days), while only 1.5% (757/51,004) of adolescents vaccinated in 2022 received a regular dosing interval (28 days or more).

**Fig. 3:**
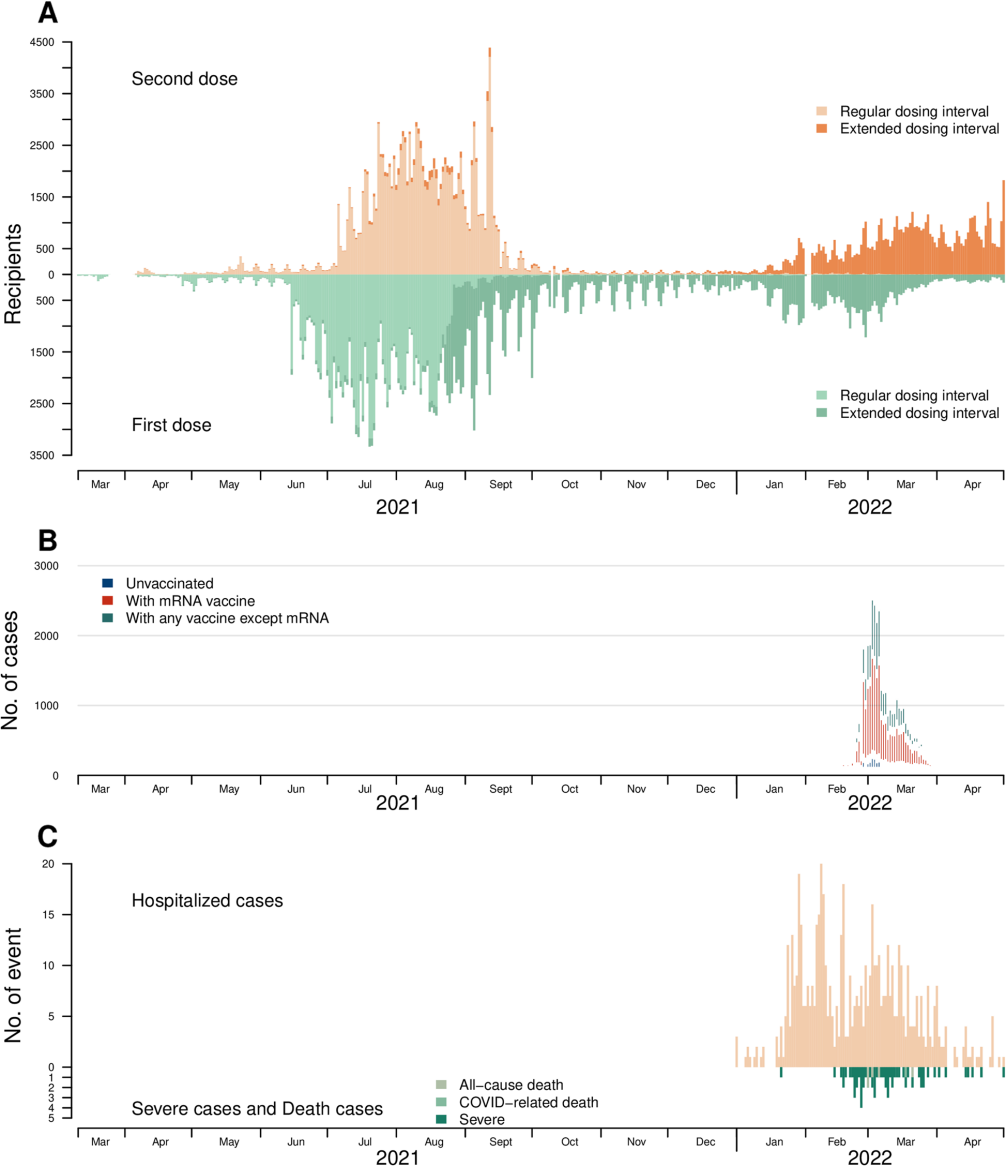
Graphical description of the modelling approach used to calculate vaccine effectiveness (VE). The risk of infection is assumed to increase with increasing time since second dose vaccination because the duration of protection is limited (i.e., VE wanes). VE is estimated from the relative risk of infection at the beginning of the period of protection versus the day at which protection wanes to VE = 0% (end day of VE waning)

Hong Kong maintained low COVID-19 incidence for most of 2020–2021 [33] but experienced a large Omicron BA.2 wave (Hong Kong’s “fifth wave”) in early 2022. Between 1 January and 30 April, there were 35,759 COVID-19 cases among adolescents aged 12–17 years (Fig. 3), among whom there were 600 hospitalizations, 5 all-cause deaths, and 1 COVID-related death. The 35,759 COVID-19 cases included 13,144 who had received their primary mRNA (2-dose) series, 3495 unvaccinated adolescents, and 19,120 who had received other vaccines or combinations of mRNA and other vaccines. We restricted this analysis to those who received mRNA, only, giving a final sample of 195,835 adolescents who had completed their primary vaccination series, including 13,144 COVID-19 cases and 182,691 non-cases. The age, sex, and dosing intervals by case status are summarized in Table S2-3.

### Comparison of infection risk for extended versus regular dosing intervals

**Fig. 4:**
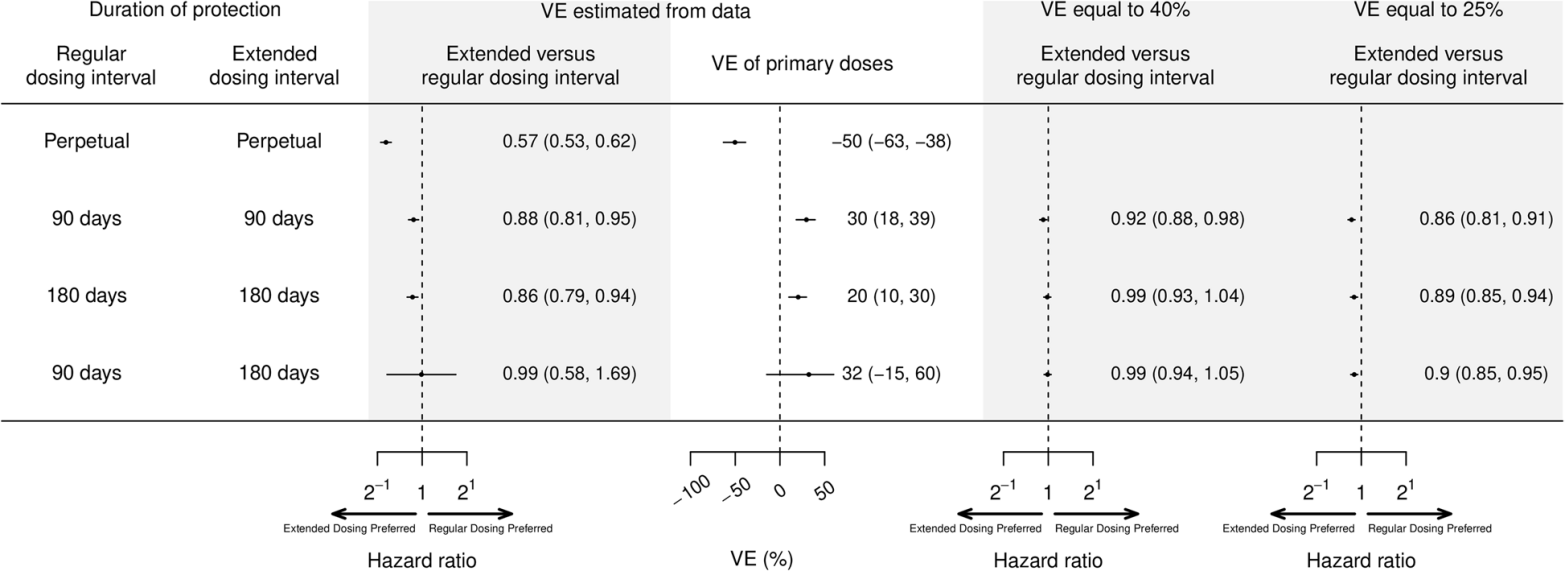
The hazard ratio (HR) of infection for extended versus regular dosing intervals, estimated from the Hong Kong Center of Health Protection data by a proportional hazard model using a calendar time scale. HRs were estimated under different assumptions about the vaccine effectiveness (VE) of primary series at the vaccination date (VE estimated from the data, VE = 40% and VE = 25%) and the duration of protection (days from second dose until protection wanes to VE = 0%) for regular and extended dosing intervals

Figure 4 summarizes the results of the analysis based on Cox proportional hazards model using a calendar time scale. When waning was assumed to be perpetual (i.e., no upper bound to the duration of waning VE), the hazard ratio (HR) comparing the risk of infection for an extended versus regular dosing interval was 0.57 (95% confidence interval (CI) 0.53, 0.62), corresponding to a VE estimate for primary series vaccination of − 50% (95% CI − 63%, − 38%). However, when we fixed the duration of protection to 90 or 180 days with zero protection thereafter, there were more modest differences in infection risk between the two dosing intervals, with HR = 0.88 (95% CI 0.81, 0.95) at 90 days and 0.86 (95% CI 0.79, 0.94) at 180 days, corresponding to VE estimates of 20% (95% CI 10%, 30%) and 32% (− 15%, 60%), respectively. When waning was shorter for the regular dose group (90 days versus 180 days for the extended group), there was no difference in infection risk between dosing intervals (HR = 0.99, 95% CI 0.58, 1.69).

Next, we fixed primary series VE to 40 or 25% (Fig. 4) and re-estimated the HRs. When primary series VE was assumed to be 25%, the risk of infection in the extended dosing interval group remained lower than the regular dosing group (HR = 0.86, 95% CI 0.81, 0.91 for 90 days; HR = 0.89, 95% CI 0.85, 0.94 for 180 days). When VE was increased to 40% there was a lower infection risk for the extended dosing interval group when protection was assumed to wane by 90 days (HR = 0.92, 95% CI 0.88, 0.98), but not by 180 days (HR = 0.99, 95%CI 0.93, 1.04).

In subgroup analyses, the infection risk among extended versus regular dosing interval groups were similar for females and males and were generally similar to the results in the primary analysis (Additional file 1: Fig. S1). In a sensitivity analysis excluding participants with extreme dosing intervals (> 100 days), the estimated HRs were similar to results in the primary analysis. When 56 days was used to define the extended dosing interval (Additional file 1: Fig. S1), HRs were similar to those obtained when the interval was 28 days when VE was fixed to 25 or 40%, but not when waning was assumed to be perpetual, and the VE was estimated from the data.

In the sensitivity analysis that restricted the analyses to the time period within 90 or 180 days after vaccination (Additional file 1: Fig. S2-3), under different assumptions on VE of primary vaccination (estimated from data, or fixed to 25 or 40%), the estimates of HR for an extended versus regular dosing interval ranged from 0.76 to 1.07. The VE estimates for primary vaccination ranged from 31 to 53%. These estimates were similar to the primary analysis except there were no negative VE estimates.

When cases and controls were matched by age and sex, and the days since vaccination of second dose was included as a covariate (waning was assumed to be perpetual) in a matching approach using a conditional logistic regression, the estimated OR for extended versus regular dosing intervals was 0.56 (95% CI 0.52, 0.62), but this OR was 0.87 (95% CI 0.80, 0.95) and 0.85 (95% CI 0.78, 0.94) when the duration of protection was assumed to be 90 and 180 days respectively. When cases and controls were matched by age and sex and days since second dose, the estimated OR for extended versus regular dosing intervals was 0.86 (95% CI 0.78, 0.94).

### Simulation study for validity of estimates

**Fig. 5:**
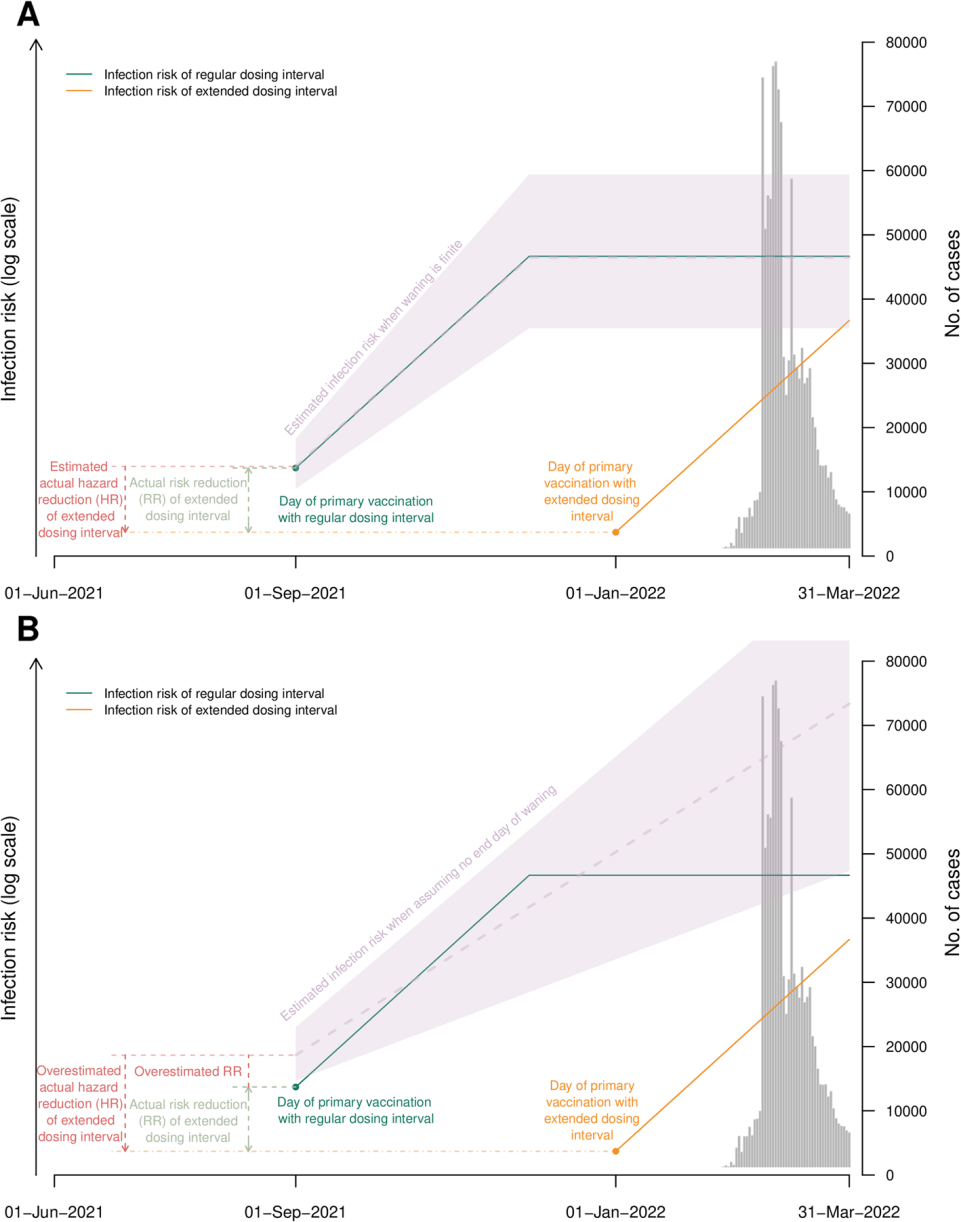
Proof-of-concept figure illustrating the impact of duration of protection (days) assumptions on estimating the hazard ratio (HR) of infection for extended versus regular dosing intervals. Panel **A** shows the scenarios with realistic assumptions about the finite duration of waning. Panel **B** show the unrealistic assumption that VE continues to wane perpetually and can allow VE < 0%. As indicated, assuming VE continued to wane without an end day would lead to overestimating the risk reduction

**Fig. 6:**
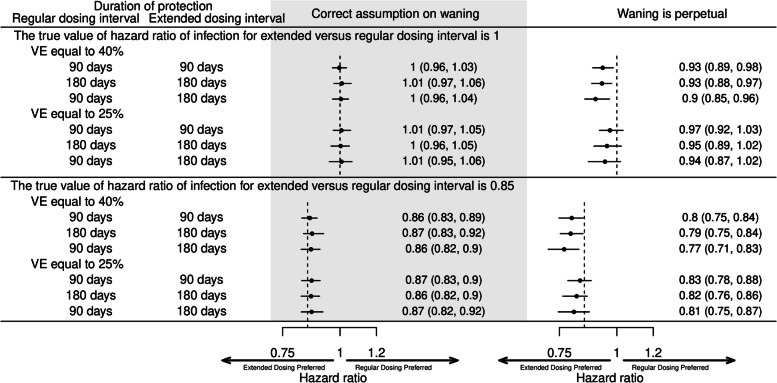
Simulation studies of the proposed proportional hazard model using a calendar time scale to explore assumption about waning VE. Two simulations were conducted in which the true value of the hazard ratio of infection comparing an extended versus regular dosing interval were set to 0.85 or 1. For each set of model parameters (vaccine effectiveness (VE) of primary series, duration of protection of regular and extended dosing intervals), 50 replications were conducted. Points and bars represent the mean, 2.5, and 97.5 percentiles of the 50 replications

We examined the impact of our assumptions about waning and primary series VE in the estimation of the HRs by constructing synthetic datasets that simulated infection outcomes since the first dose for each individual and removed those infected before the second dose (Fig. 5). The simulations showed that our proportional hazards model could recover the true HR under realistic assumptions about the duration of protection (Fig. 6); i.e., that waning has some finite values. When waning is assumed to continue perpetually, which allows protection to reduce beyond 0%, HRs were under-estimated.

**Fig. 7:**
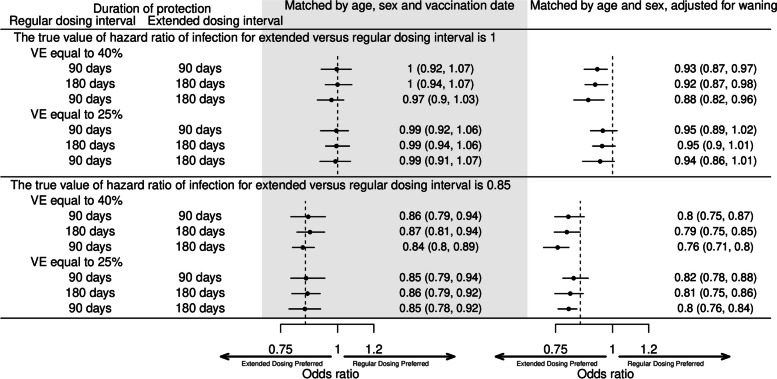
Simulation studies using a case-density matching approach. Hazard ratios (HR) were estimated using conditional logistic regression. Two simulations with setting the true value of hazard ratio of infection of extended versus regular dosing intervals to be 0.85 or 1 were conducted. For each set of model parameters (vaccine effectiveness (VE) of primary series, waning end days of regular and extended dosing intervals), 50 replications were conducted. Points and bars represent the mean, 2.5, and 97.5 percentiles of the 50 replications

We also tested the matching approach in simulated case-density datasets (Fig. 7). In the first model, the date of vaccination of second dose was included as a matching variable and the days since second dose was not adjusted for in the model. In nearly all cases and for all durations of protection assessed the OR could approximate the true values of the HRs. In the second model, the number of days since second dose was included as a covariate. When waning was assumed to continue perpetually, the recovered ORs underestimated the true HR for when VE = 40% or VE = 25%.

### Comparison of infection risk of extended versus regular dosing intervals since first dose instead of second dose based on real-world data

Based on the calendar-time proportional hazards model (Table 1), we estimated that adolescents in the extended dosing groups (including those did not receive second dose in the study period) had a higher hazard of infection than regular dosing groups in both 42–98 days (HR 1.66; 95% CI 1.07, 2.59; *p* = 0.02) and 42–70 days (HR 1.71; 95% CI 1.06, 2.77; *p* = 0.03) after first dose. In sensitivity analyses that allowing 7 days instead of 14 days for vaccine to take effects, there were still higher hazard of infection for extended dosing groups compared with regular dosing group (Table 1).

**Table 1:** Results from the calendar-time proportional hazards model to compare the hazard ratio of infection during intra-dose period for extended (28 days or more after first dose), versus regular dosing interval (21–27 days). Two policies were compared, including extended dosing interval to 84 days (on 23 December 2021), and 56 days (on June 22, 2022). Fourteen days was allowed for vaccine to take effect. In sensitivity analysis, 7 days was allowed instead

	Received second dose within 21–27 days after first dose (Reference group)	Received second dose in 28 days or more after first dose, or never received second dose
***Allowing 14 days for vaccine to take effects***		
Comparison of infection risk in 42–98 days after first dose; person-day: 1,290,204		
Number of infections; number of people	20/575	1073/37,950
Hazard ratio (95% CI; *p*-value)	1.66 (1.07, 2.59); *p* = 0.02	
Comparison of infection risk in 42–70 days after first dose; person-day: 900,311		
Number of infections	17/575	977/37,950
Hazard ratio (95% CI; *p*-value)	1.71 (1.06, 2.77); *p* = 0.03	
***Allowing 7 days for vaccine to take effects***		
Comparison of infection risk in 35–91 days after first dose; person-day: 1,508,630		
Number of infections	28/587	1537/40,164
Hazard ratio (95% CI; *p*-value)	1.54 (1.06, 2.24); *p* = 0.02	
Comparison of infection risk in 35–63 days after first dose; person-day: 1,508,630		
Number of infections; number of people	23/587	1343/40,164
Hazard ratio (95% CI; *p*-value)	1.54 (1.02, 2.32); *p* = 0.04	

### Simulation study to compare the risk of infection since first dose instead of second dose

**Fig. 8:**
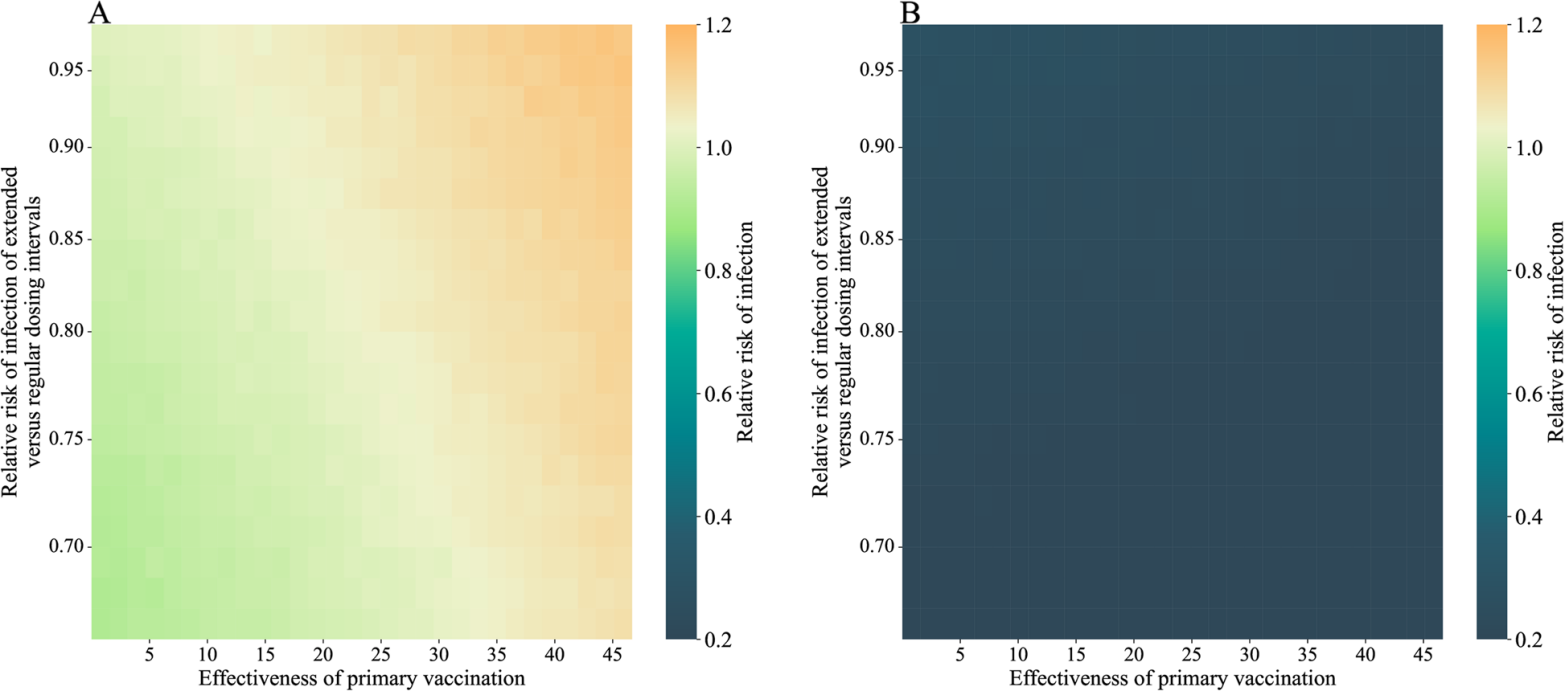
Simulation studies comparing the relative risk of infection for extended versus regular dosing intervals since the first dose rather than the second dose. Panels **A** and **B** show the relative risk of infection since first dose and second dose, respectively. In this simulation, regular and extended dosing intervals were defined as 21 and 56 days, respectively. For each set of parameters, 100 replications on 20,000 participants with equal proportions of individuals receiving extended and regular dosing intervals were simulated. The mean relative risk of infection of 100 replications was recorded. The waning end day was set to 90 days for both regular and extended dosing intervals. The daily risk of infection was set to be proportional to the epidemic curve in the fifth wave in Hong Kong

In a simulation study that the baseline risk of infection was varying and set to be proportional to the observed infection rate in January 1 to April 30, 2022, in Hong Kong (Fig. 8), the RR of infection since second and first dose ranged from 0.16 to 0.28, and from 0.91 to 1.16, respectively when the duration of protection for regular and extended dosing intervals were 90 days. The simulation results were similar if the duration of protection for regular and extended dosing intervals were 90 days and 180 days respectively (Additional file 1: Fig. S4). If the baseline risk of infection was set to be constant, the RR of infection since second and first dose ranged from 0.34 to 0.56, and from 0.85 to 1.02, respectively when the duration of protection for regular and extended dosing intervals were 90 days (Additional file 1: Fig. S5). The simulation results were similar if the duration of protection for regular and extended dosing intervals were 90 days and 180 days respectively (Additional file 1: Fig. S6).

## Discussion

In this study, we conducted a comprehensive analysis to estimate the relative risk of infection among adolescents receiving a primary series of mRNA vaccine with regular (21–27 days) or extended (≥ 28 days) dosing intervals in Hong Kong. Overall, we estimated that the risk of infection among adolescents receiving their primary vaccination series was lower for those receiving an extended versus regular dosing interval (HR ranged from 0.86 to 0.99), based on reasonable assumptions, and corresponding to an absolute increase in VE of 1 to 14%. Furthermore, we estimated that there was an increased risk during the inter-dose period for adolescents in extended dosing groups.

Although the definition of an extended dosing interval varied (ranging from ≥ 28 to ≥ 84 days), our estimates were consistent with previous studies that suggest moderate benefit of extended dosing intervals. Studies among adolescents [13] and healthcare workers in Canada [34], and a study among individuals aged 50 + in the UK [35] all observed only a 5–10% absolute increase in VE when the dosing interval was extended. Other studies, including a UK household [36] and healthcare workers study [37] found no difference. Only one study has observed a much higher VE (28% absolute increase) after an extended dosing interval, which was the paper by Lai et al. from Hong Kong [11].

The higher VEs reported in the literature refer to infection risk after the second vaccination dose. Here, we estimated that the infection risk during the inter-dose period was significantly increased, among adolescents in the extended dosing group.

The VE is not appreciably improved by an extended dosing interval, and there was potential increased risk of infection during the inter-dose period. Taken together, the decision to recommend delaying second doses or not could also consider other factors such as [1] whether the population immunity achievable from administering a single dose to more people more quickly exceeds what could be achieved by administering two doses to a smaller group [38, 39]; [2] the duration of the extra protection; [3] the potential for near-future outbreaks requiring rapid distribution of vaccine to provide protection; [4] the reduction in risk of severe adverse events following vaccination, such as myocarditis, balanced with the risk of these events from infections [40].

We also explored alternative approaches for estimating the HR of infections for extended versus regular dosing intervals. We observed that adjusting for waning by simply including the days since second dose as a linear covariate could underestimate the HR (overestimate VE), as this approach implicitly assumes that the waning never ends. More robust estimates can be recovered when the duration of waning has an upper limit. This problem persists when a matched approach is used. Thus, we suggest that the previously reported 28% higher VE for extended versus regular dosing intervals [11] was likely an overestimate. Although we focused on estimating relative VE for extended versus regular dosing intervals, incorrect assumptions about waning could also affect the estimation of absolute VE and booster dose VE.

It should be noted that VE estimates for the primary series in this study should be interpreted with caution, as they are projected from waning VE, and no unvaccinated individuals were included. The validity for VE estimated from this method and the potential of depletion of susceptible bias has been discussed in previous studies [41, 42]. In brief, the estimate was unbiased when there was no VE waning, and underestimated the degree of waning when there was VE waning. Hence, we also adopted the approach that estimating the relative VE for primary series vaccination (compared with the first day of vaccination) and the hazard ratio of infection for extended versus regular dosing intervals.

There were some limitations in our study. First, as an observational study, we cannot rule out the potential for unidentified confounders, such as unmeasured differences that may exist among individuals who chose regular or extended dosing intervals or were eligible for vaccination early and thus received a regular dosing interval. Second, information on case variants was unavailable, but the predominant variant during our study period was Omicron BA.2 [43]. Finally, in the simulations, we used the infection rate observed from January 1 to April 30, 2022, as an input of the model and simulated the outcomes to the observed data (using same covariates) to generate synthetic datasets. However, some milder or asymptomatic cases could be missed despite the compulsory reporting of cases implemented in Hong Kong [44]. Also, it is possible that the decision of vaccination, and choice of an extended or regular dosing interval may change depending on the epidemic trajectory [45, 46].

## Conclusions

Our analysis of population-based case and vaccination data found that the VE for extended BNT162b2 dosing intervals was 1–14% higher than for regular intervals, under reasonable assumptions regarding duration of VE waning and the VE of primary vaccination. Our simulation study suggested that unreasonable assumptions may overestimate the extra protection afforded by extended dosing intervals and we recommend that any VE analysis carefully consider how waning is parameterized. Although the additional protection afforded by an extended dosing interval may be limited, other public health considerations may drive recommendations to extend dosing intervals.

## Supplementary Information

Additional file 1: Fig. S1. Sensitivity analysis of the hazard ratio (HR) of infection for extended versus regular dosing interval. Fig. S2. Sensitivity analysis of the hazard ratio (HR) of infection for extended versus regular dosing intervals restricted to the time period within 90 days after vaccination. Fig. S3. Sensitivity analysis of the hazard ratio (HR) of infection for extended versus regular dosing intervals restricted to the time period within 180 days after vaccination. Fig. S4. Simulation studies to compare the relative risk of infection of extended verses regular dosing interval, since the first dose instead of second dose, setting the end day of waning to be 90 and 180 days for both regular and extended dosing intervals respectively. Fig. S5. Simulation studies to compare the relative risk of infection of extended verses regular dosing interval, since the first dose instead of second dose, setting the risk of infection to be constant. Fig. S6. Simulation studies to compare the relative risk of infection of extended verses regular dosing interval, since the first dose instead of second dose, setting the risk of infection to be constant, and the end day of waning to be 90 and 180 days for both regular and extended dosing intervals respectively. Table S1. Summary of previous studies that explored the impact on extended versus regular dosing intervals. Table S2: Characteristics of case and non-cases in our study. Table S3: Intervals of vaccination to infection between extended and regular dosing group.

## Data Availability

Access to the case, hospitalization and vaccination data from the electronic medical record system managed by the Hospital Authority and other databases by the Centre for Health Protection in Hong Kong is subject to the approval from the two agencies.

## Abbreviations

HR: Hazard ratio
OR: Odds ratio
VE: Vaccine effectiveness
