# Supplementary material for: Evaluating the impact of extended dosing intervals on mRNA COVID-19 vaccine effectiveness in adolescents
Source: BMC Med. 2024 Sep 12;22:384. doi: 10.1186/s12916-024-03597-4 (PMC11396738; doi:10.1186/s12916-024-03597-4)
Supplement: Supplementary file 1 — Additional file 1: Fig. S1. Sensitivity analysis of the hazard ratio (HR) of infection for extended versus regular dosing interval. Fig. S2. Sensitivity analysis of the hazard ratio (HR) of infection for extended versus regular dosing intervals restricted to the time period within 90 days after vaccination. Fig. S3. Sensitivity analysis of the hazard ratio (HR) of infection for extended versus regular dosing intervals restricted to the time period within 180 days after vaccination. Fig. S4. Simulation studies to compare the relative risk of infection of extended verses regular dosing interval, since the first dose instead of second dose, setting the end day of waning to be 90 and 180 days for both regular and extended dosing intervals respectively. Fig. S5. Simulation studies to compare the relative risk of infection of extended verses regular dosing interval, since the first dose instead of second dose, setting the risk of infection to be constant. Fig. S6. Simulation studies to compare the relative risk of infection of extended verses regular dosing interval, since the first dose instead of second dose, setting the risk of infection to be constant, and the end day of waning to be 90 and 180 days for both regular and extended dosing intervals respectively. Table S1. Summary of previous studies that explored the impact on extended versus regular dosing intervals. Table S2: Characteristics of case and non-cases in our study. Table S3: Intervals of vaccination to infection between extended and regular dosing group. [file 12916_2024_3597_MOESM1_ESM.docx]

**Additional File 1**

**Evaluating the impact of extended dosing intervals on mRNA COVID-19 vaccine effectiveness in adolescents**

Tim K. Tsang^1,2^, Sheena G. Sullivan^3,4^, Yu Meng^1^, Francisco Tsz Tsun Lai^2,5^, Min Fan^5^, Xiaotong Huang^1^, Yun Lin^1^, Liping Peng^1^, Chengyao Zhang^1,^, Bingyi Yang^1^, Kylie E. C. Ainslie^1,6^, Benjamin J. Cowling^1,2^

**Affiliations:**

1. WHO Collaborating Centre for Infectious Disease Epidemiology and Control, School of Public Health, Li Ka Shing Faculty of Medicine, The University of Hong Kong, Hong Kong Special Administrative Region, China
2. Laboratory of Data Discovery for Health Limited, Hong Kong Science and Technology Park, New Territories, Hong Kong Special Administrative Region, China
3. School of Clinical Sciences, Monash University, Melbourne, Australia
4. Department of Epidemiology, University of California, Los Angeles, USA
5. Centre for Safe Medication Practice and Research, Department of Pharmacology and Pharmacy, Li Ka Shing Faculty of Medicine, The University of Hong Kong, Hong Kong Special Administrative Region, China
6. Centre for Infectious Disease Control, National Institute for Public Health and Environment (RIVM), Bilthoven, the Netherlands

**Corresponding author:**

Tim K. Tsang, School of Public Health, Li Ka Shing Faculty of Medicine, The University of Hong Kong, 7 Sassoon Road, Pokfulam, Hong Kong.

Tel: +852 3917 9715; Fax: +852 3520 1945; email: [timtsang@connect.hku.hk](mailto:timtsang@connect.hku.hk)

Benjamin J Cowling, School of Public Health, The University of Hong Kong, 7 Sassoon Road, Pokfulam, Hong Kong.

Tel: +852 3917 6711; Fax: +852 3520 1945; email: bcowling@hku.hk

**Simulation model for the infection since first dose for individuals with extended and regular dosing intervals.**

**Overview**

The main interest is to estimate the protection of extended dosing intervals comparing to regular dosing intervals. The difficulties are:

1. The regular schedule was implemented before the extended schedule. Therefore, extended schedule appeared to be better due to waning of vaccine effectiveness (VE) of primary vaccination.
2. The risk of infection was varying.

To overcome this, we developed a simulation model for the risk of infection since first dose, and to determine the validity of different approaches to estimate the RR of infection of extended verses regular dosing intervals, accounting for factors such as waning VE.

**Model overview**

Denote $[T_{s},T_{e}]$ as the start and the end date of the study. We defined the study cohort as all adolescents aged 12-17, and vaccinated with 2-dose BNT in the study period in Hong Kong.

For each individual *i*, we introduce a vector $(y_{i},t_{i},t_{1i},t_{2i},t_{3i},g_{i},a_{i},s_{i})$, where $y_{i}$ is infection status and $t_{i}$ is the infection time, $t_{1i},t_{2i}$ are the day of vaccination of the first and second dose respectively (added 14 days to the actual vaccination time to allow for the delay for vaccination to be effective), $g_{i}$, $a_{i}$ and $s_{i}$ are the type of intervals (1: extended, 0: regular), age and sex of the individuals.

In our model, we assumed that participant could only be infected once during the outbreak. Also, 14 days were allowed for the delay for receipt of vaccine and the effect took off.

**Infection model**

We use the COVID-19 case number during the study period (denoted as *P*) to as a proxy of the unscaled incidence of infection of COVID-19.

The probability of infection at time t for individual *i* is:

$$logit\left( P\left( t | t_{1i},t_{2i},g_{i},a_{i},s_{i} \right) \right)=logit\left( \phi*P_{t} \right)+\beta_{1}*g_{i}+\beta_{2}*a_{i}+\beta_{3}*s_{i}+\beta_{4}*f_{w}\left( t-t_{2i} \right)$$

where $\phi$ is the scale parameter for the risk of infection, $\beta_{1}$ is effect associated with extended dosing intervals, compared to regular dosing intervals, $\beta_{2}$ and $\beta_{3}$ are the effects associated with age and sex respectively. $\beta_{4}$ is the VE of primary vaccination, and $f_{w}(.)$ is the function describing the waning over time.

We set

$$f_{w}\left( t \right)=\frac{t}{t_{max}}*I\left( t\in\left[ 0,t_{max} \right] \right)+1*I\left( t\notin[0,t_{max}] \right)$$

Hence, after receiving the second dose, the waning is proportional to the days since second dose, and reach the maximum when t equal to $t_{max}$, which is the day when waning end, and $exp(\beta_{4})$ is the odds ratio of infection for first dose verses second dose. Assuming first dose provided no protection, $100*(1-\exp\left( -\beta_{4} \right))$ is the VE of primary vaccination.

**Simulation study**

For the simulation about validating estimation approach, we used above simulation model to simulation the infection outcome since the first dose. To mimic the selection process of real data, all individuals infected before the receipt of second dose were excluded. Then, we applied our proposed proportional hazard using a calendar time scale ([22](#_ENREF_22)), and also the matching approach with conditional logistic regression ([11](#_ENREF_11)) to obtain the parameter estimates. For each model parameter and estimation approach, 50 replications were performed. The mean, 2.5 and 97.5 percentile for the estimates from 50 replications were reported.

For the simulation study of the comparing the infection risk of extended and regular dosing interval since the first dose, instead of second dose, we defined that regular and extended dosing interval were defined as 21 and 56 days respectively. For each set of parameters, 100 replications on 20000 participants, with equal proportion of individual received extended and regular dosing intervals, were simulated. Then mean of the relative risk of infection since first dose and second dose of 100 replications were recorded. The end day of waning were set to be 90 days for both regular and extended dosing intervals. Constant and varying risk during epidemics referred the daily risk of infection were constant, or set to be proportion to the epidemic curve in the fifth wave in Hong Kong respectively.

**SUPPLEMENTARY FIGURE**

**Fig. S1.** Sensitivity analysis of the hazard ratio (HR) of infection for extended versus regular dosing interval. It was estimated by a proportion hazard model using a calendar time scale. HRs were estimated under different assumptions on the vaccine effectiveness (VE) of primary series, end days of waning for regular and extended dosing interval. Sex-specific analyses and sensitivity analyses including using 56 days instead of 28 days as cutoff of extended dosing intervals, and excluding participants with extreme intervals (>100 days) were performed.

**Fig S2.** Sensitivity analysis of the hazard ratio (HR) of infection for extended versus regular dosing intervals restricted to the time period within 90 days after vaccination. It was estimated from the Hong Kong Center of Health Protection data by a proportional hazard model using a calendar time scale. The analysis is using the same models in Figure 4. HRs were estimated under different assumptions about the vaccine effectiveness (VE) of primary series at the vaccination date (VE estimated from the data, VE=40% and VE=25%) and the duration of protection (days from second dose until protection wanes to VE=0%) for regular and extended dosing intervals.

**Fig S3.** Sensitivity analysis of the hazard ratio (HR) of infection for extended versus regular dosing intervals restricted to the time period within 180 days after vaccination. It was estimated from the Hong Kong Center of Health Protection data by a proportional hazard model using a calendar time scale. The analysis is using the same models in Figure 4. HRs were estimated under different assumptions about the vaccine effectiveness (VE) of primary series at the vaccination date (VE estimated from the data, VE=40% and VE=25%) and the duration of protection (days from second dose until protection wanes to VE=0%) for regular and extended dosing intervals.

**Fig S4.** Simulation studies to compare the relative risk of infection of extended verses regular dosing interval, since the first dose instead of second dose, setting the end day of waning to be 90 and 180 days for both regular and extended dosing intervals respectively. The figure setting is the same as the Figure 8.

**Fig S5.** Simulation studies to compare the relative risk of infection of extended verses regular dosing interval, since the first dose instead of second dose, setting the risk of infection to be constant. The risk of infection was set to be proportional to the real data from January to April 2022 in Figure 8. The figure setting is the same as the Figure 8.

**Fig S6.** Simulation studies to compare the relative risk of infection of extended verses regular dosing interval, since the first dose instead of second dose, setting the risk of infection to be constant, and the end day of waning to be 90 and 180 days for both regular and extended dosing intervals respectively. The figure setting is the same as the Figure 8.

**Table S1:** Summary of previous studies that explored the impact on extended versus regular dosing intervals

| Author, year | Country/  region | Study type | Age group | Vaccine type | Grouping in estimating impact (based on outcome mearues) | Outcome measures | Estimates of impact and conclusion |
| --- | --- | --- | --- | --- | --- | --- | --- |
| Chantasrisawad, 2023  ([9](#_ENREF_9)) | Thailand | RCT | School-aged children (5-11 years) | BNT162b2 | 8-week VS 3-week interval between first and second dose | 1. Neutralization test (NT) against the Omicron variant, surrogate virus NT (sVNT; BA.1, % inhibition)  2. Pseudovirus NT (BA.2, the half-maximal inhibition dilution or ID50)  3. Quantitative anti-spike-receptor binding domain immunoglobulin G enzyme-linked immunosorbent assay  Index: Geometric means ratio (GMR) | Extended dosing intervals could enhance the antibody response.  1. sVNT: 3.0 (95% CI:2.4, 3.8)  2. pVNT: 5.7 (95% CI:3.5, 9.3) |
| Andrews, 2022  ([47](#_ENREF_47)) | England | TND | 80 years of age or older | ChAdOx1-S, BNT162b2 | ≤28 days (≤4 weeks) or ≥56 days (≥8 weeks) | VE against hospitalization | The results showed lower VE among participants with a short interval (≤4 weeks) than among those with an extended interval (≥8 weeks) between doses in the latest follow-up periods (≥20 weeks after the second dose).  However, confidence intervals were wide and overlapping (see Figure S5). |
| Ionescu, 2023  ([13](#_ENREF_13)) | Canada | TND | Adolescents aged 12-17 years | BNT162b2 | 21-34d (3-4weeks),  35-48d (5-6weeks),  49-55d (7weeks),  56-62d (8weeks),  63-83d (9-11weeks), 84+d (12+weeks),  56+d (8+weeks) VS unvaccinated | VE = (1– adjusted odds ratio) ×100% | A longer interval between first and second doses improved Omicron protection, a preferred 8-week versus the manufacturer-specified 3-week interval between doses was recommended.  1. Delta dominant (Epi-weeks 36-47)  21-34d (3-4weeks): 93.9 (92.3, 95.2)  35-48d (5-6weeks): 95.8 (94.9, 96.6)  49-55d (7weeks): 95.5 (94.4, 96.5)  56-62d (8weeks): 94.8 (93.6, 95.7)  63-83d (9-11weeks): 96.5 (95.7, 97.1)  84+d (12+weeks): 95.1 (92.0, 97.0)  56+d (8+weeks): 95.8 (95.1, 96.3)  2. Delta-Omicron transition (Epi-weeks 48-50)  21-34d (3-4weeks): 81.6 (78.4, 84.2)  35-48d (5-6weeks): 80.6 (77.8, 83.0)  49-55d (7weeks): 80.8 (77.4, 83.6)  56-62d (8weeks): 80.4 (77.3, 83.1)  63-83d (9-11weeks): 82.4 (79.9, 84.6)  84+d (12+weeks): 86.1 (80.3, 90.2)  56+d (8+weeks): 82.5 (80.4, 84.4)  3. Omicron dominant (Epi-weeks 51-17)  21-34d (3-4weeks): 37.7 (32.1, 42.8)  35-48d (5-6weeks): 39.4 (34.3, 44.2)  49-55d (7weeks): 40.7 (35.3, 45.8)  56-62d (8weeks): 43.0 (37.9, 47.7)  63-83d (9-11weeks): 42.3 (37.5, 46.7)  84+d (12+weeks): 55.4 (49.6, 60.5)  56+d (8+weeks): 44.5 (40.3, 48.5) |
| Nasreen, 2023  ([48](#_ENREF_48)) | Canada | TND | Adults aged ≥18 years | mRNA vaccines  (mRNA; BNT162b2, Pfizer-BioNTech Comirnaty and  mRNA-1273, Moderna Spikevax) | 21–34 days,  35–55 days,  56–83 days,  ≥84 days VS Unvaccinated | VE = (1- OR) ×100% | A slight difference in VE between short and extended dosing intervals for mRNA vaccines, and 95 % CIs overlapped.  1. Dosing interval 21-34 days:  0-6 day after dose 2: 94 (47, 99),  7–55 days after dose 2: 91 (85, 95),  56–111 days after dose 2: 98 (97, 98)  ≥112 days after dose 2: 97 (93, 99)  2. Dosing interval 35-55 days:  0-6 day after dose 2: 94 (47, 99),  7–55 days after dose 2: 98 (94, 99),  56–111 days after dose 2: 99 (98, 100)  ≥112 days after dose 2: 97 (95, 98)  3. Dosing interval 56-83 days:  0-6 day after dose 2: 94 (89, 96)  7–55 days after dose 2: 99 (97, 99)  56–111 days after dose 2: 99 (98, 100)  ≥112 days after dose 2: 97 (96, 99)  4. Dosing interval ≥84 days  0-6 day after dose 2: 93 (79, 97)  7–55 days after dose 2: 98 (94, 99)  56–111 days after dose 2: 99 (95, 100)  ≥112 days after dose 2: 99 (97, 100) |
| Skowronski, 2022  ([12](#_ENREF_12)) | Canada | TND | Adults aged ≥18 years | mRNA vaccine and/or ChAdOx1 | Longer (7–8-week), manufacturer-specified (3–4-week) intervals | VE against infection or hospitalization (any two mRNA vaccines)  = (1 – adjusted OR) × 100% | A 7–8-week interval between first and second doses improved mRNA VE and maybe the optimal schedule outside periods of intense epidemic surge.  1. SARS-CoV-2 Infection risk  21-34d: 93.9 (92.3, 95.2)  35-48d: 95.8 (94.9, 96.6)  49-62d: 95.5 (94.4, 96.5)  63-63d: 94.8 (93.6, 95.7)  84-111d: 96.5 (95.7, 97.1)  112+d: 95.1 (92.0, 97.0)  49+d: 95.8 (95.1, 96.3)  2. Hospitalization risk  21-34d: 93.9 (92.3, 95.2)  35-48d: 95.8 (94.9, 96.6)  49-62d: 95.5 (94.4, 96.5)  63-63d: 98 (98, 98)  84-111d: 95 (94, 96)  112+d: 93 (89, 95)  49+d: 98 (97, 98) |
|  |  |  |  |  |  | VE against infection or hospitalization (two ChAdOx1 vaccines)  = (1 – a OR) × 100% | 1. SARS-CoV-2 Infection risk  21-34d (3-4weeks): 81 (58, 91)  35-48d (5-6weeks): 76 (67, 82)  49-62d (7-8weeks): 75 (73, 77)  63-63d (9-11weeks): 74 (71, 77)  84-111d (12-15weeks): 62 (53, 70)  112+d (16+weeks): 73 (33, 89)  49+d (7+weeks): 74 (72, 76)  2. Hospitalization risk  21-34d (3-4weeks): NE  35-48d (5-6weeks): 94 (59, 99)  49-62d (7-8weeks): 96 (93, 97)  63-63d (9-11weeks): 96 (93, 97)  84-111d (12-15weeks): 93 (72, 98)  112+d (16+weeks): NE  49+d (7+weeks): 95 (94, 97) |
|  |  |  |  |  |  | VE against infection or hospitalization (two BNT162b2 vaccines)  = (1 – adjusted OR) × 100% | 1. SARS-CoV-2 Infection risk  21-34d (3-4weeks): 84 (83, 86)  35-48d (5-6weeks): 83 (82, 84)  49-62d (7-8weeks): 90 (89, 90)  63-63d (9-11weeks): 89 (89, 90)  84-111d (12-15weeks): 87 (86, 87)  112+d (16+weeks): 87 (86, 89)  49+d (7+weeks): 89 (89, 90)  2. Hospitalization risk  21-34d (3-4weeks): 97 (93, 98)  35-48d (5-6weeks): 96 (95, 98)  49-62d (7-8weeks): 99 (98, 99)  63-83d (9-11weeks): 98 (97, 98)  84-111d (12-15weeks): 95 (94, 96)  112+d (16+weeks): 91 (87, 94)  49+d (7+weeks): 98 (97, 98) |
|  |  |  |  |  |  | VE against infection or hospitalization (two mRNA-1273 vaccines)  = (1 – adjusted OR) × 100% | 1. SARS-CoV-2 Infection risk  21-34d (3-4weeks): 89 (87, 91)  35-48d (5-6weeks): 85 (83, 86)  49-62d (7-8weeks): 91 (91, 92)  63-83d (9-11weeks): 91 (91, 92)  84-111d (12-15weeks): 84 (83, 86)  112+d (16+weeks): 88 (84, 90)  49+d (7+weeks): 90 (90, 91)  2. Hospitalization  21-34d (3-4weeks): 96 (91, 98)  35-48d (5-6weeks): 94 (90, 96)  49-62d (7-8weeks): 99 (98, 99)  63-63d (9-11weeks): 98 (98, 99)  84-111d (12-15weeks): 95 (93, 97)  112+d (16+weeks): 95 (88, 98)  49+d (7+weeks): 98 (97, 98) |
| Canderan, 2023  ([49](#_ENREF_49)) | Canada | Observational | Healthcare workers  (43±13) | BNT162b2 | <35-days,  35-42-days,  >42-days | 1. Ancestral SARS-CoV-2 anti-Spike and anti-RBD IgG titers  2. Ancestral SARS-CoV-2 anti-Spike and anti-RBD IgA and IgM titers  3. Neutralizing titers against SARS-CoV-2 ancestral and beta variant  4 anti-RBD IgG secreting memory B cells (at 3 months) | Delaying the second dose beyond 42 days can potentiate and prolong the humoral response against ancestral and Beta variants of SARS-CoV-2 up to 9 months postvaccination.  1. Ancestral SARS-CoV-2 anti-Spike IgG titers  >42-days VS <35-days: mean 2.14 (1.86–2.62) VS 1.89 (1.50–2.32), *P*<0.0001 (6-9 months)  2. Ancestral SARS-CoV-2 anti-RBD IgG titers  >42-days VS <35-days: mean 2.65 (2.08–2.88) VS 2.14 (1.66–2.65), *P*=0.0002 (3 months)  >42-days VS <35-days: mean 0.79 (0.63–1.23) VS 0.67 (0.45–0.88), *P*=0.0010 (6-9 months)  3. Ancestral SARS-CoV-2 anti-Spike and anti-RBD IgA and IgM titers  Significant waning in IgA titers during longer intervals between doses.  Peak anti-RBD IgA titers were significantly lower in the 35-42-days and >42-days groups (compared to <35-days group) (3 weeks)  4. neutralizing titers against SARS-CoV-2 ancestral and beta variant  neutralizing titers against ancestral SARS-CoV-2 were highest in the >42-days group (all timepoints post-second dose)  <35-days VS >42-days: log mean 4.38(3.69–5.08) VS 5.08 (4.38–5.77), *P*<0.0001 (3 months)  <35-days VS >42-days: log mean 2.30(1.61–3.00) VS 3.00 (2.30–3.69), *P* = 0.0007 (6-9 months)  5. neutralizing titers against SARS-CoV-2 beta variant  the >42-days group had significantly higher neutralizing capacity  6. anti-RBD IgG secreting memory B cells  ≤42days VS >42days: 0.76 (SD 0.68) VS 0.305 (SD 0.282), *P*=0.016 |
| Lai, 2023  ([11](#_ENREF_11)) | Hong Kong | Observational (nested case–control study) | Children and adolescents aged 5-17 | BNT162b2 | 8 weeks, ≥28 days (extended intervals), 21-27 days (regular intervals) | Adjusted odds ratio of infections | An extended dosing interval for mRNA vaccines should be considered for children and adolescents to reduce the risk of myocarditis.  1. extended intervals VS regular intervals  Covid-19 (SARS-CoV-2) infection  0.718(0.619, 0.833)  Covid-19-related hospitalization  0.743(0.338, 1.636)  2. 28–55 days VS regular intervals  0.848(0.715, 1.006)  3. 56–83 days VS regular intervals  0.652(0.485, 0.877)  4. 84 days or above VS regular intervals  0.540(0.435, 0.672)  5. 8 weeks VS regular intervals  0.565(0.456, 0.700) |
| Hall, 2022  ([10](#_ENREF_10)) | Canada | Observational (cohort study) | Health care workers (median40.9(IQR34.5–52.5)) | BNT162b2 | Standard (3- to 6-week) and delayed (8- to 16-week) intervals | 1. Anti-receptor-binding domain antibody titers  2. plaque reduction neutralization test (PRNT50) and PRNT90 titers  3. Spike-specific polyfunctional CD4+ and CD8+ T cells expressing interferon-γ and interleukin-2 | Anti-receptor-binding domain antibody titers were significantly enhanced in the delayed-interval group, compared to the standard-interval group.  1. anti-RBD antibody titer  delayed-interval VS standard-interval: median 10816 U/ml (IQR 6,642–16,695) VS 3420 U/ml (IQR, 1,792–7,765), P<0.0001  2. PRNT50 and PRNT90 titers  Geometric mean PRNT50 titers were significantly higher in the delayed-interval group against wild-type, Alpha, Beta and Delta (data not shown)  3. Spike-specific polyfunctional CD4+ and CD8+ T cells expressing interferon-γ and interleukin-2 generally not significantly different |
| Català, 2021  ([50](#_ENREF_50)) | European countries | Simualtion | All ages | Oxford/AstraZeneca vaccine, Moderna vaccine,  Pfizer/BioNTech vaccine | Dose intervals ranging from 3 to 12 weeks | 1. mortality  2. hospitalization  3. public health restrictions | The choice of interval between doses depends on expected vaccine availability and first-dose efficacy, with 12-week intervals preferred over shorter intervals in most realistic scenarios. |
| Imai, 2023  ([3](#_ENREF_3)) | England | Simulation | Individuals aged 25 years or older | BNT162b2, ChAdOx1 nCoV-19 | Dose interval from 3 to 12 weeks | Numbers of daily infections, hospital admissions, and deaths | Delaying the interval between the first and second COVID-19 vaccine doses from 3 to 12 weeks reduced COVID-19 infections, hospital admissions and deaths. |
| Liu, 2022  ([4](#_ENREF_4)) | European countries | Simulation | All ages | AZD1222 | Both fixed dosing intervals at 4, 8, 12, 16, and 20 weeks and  dose-specific intervals that prioritise specific doses for certain age groups | Country-level daily reported COVID-19 mortality | A four-week fixed dosing interval may incur more deaths, and benefit-risk ratios were the highest for fixed dosing intervals of 8-12 weeks |
| Souto Ferreira, 2022  ([51](#_ENREF_51)) | Brazil | Simulation | Children and teenagers (0–19), adults (20–59), older adults (60+) | CoronaVac  AZD1222  BNT162b2 | 3 weeks, 7 weeks and 12 weeks | 1. Acquiring the disease  2. developing symptoms  3. developing severe symptoms (i.e., leading to hospitalization)  4. death (key outcome) | The best strategy depends on the interplay between the vaccine production rate and the single-dose relative efficacy.  1. relative efficacy of the first dose below approximately 45%: regardless of the vaccine production rate, the best strategy to reduce mortality is to complete the two-dose scheme 3 weeks after the initial dose.  2. the production rate is low (approximately below 0.2% of doses/population-day) and relative efficacy > 45%: the best time window to increasingly larger periods, and converges to the maximum interval of 12 weeks  3. relative efficacy > 45% and increasing production rates: a non-linear transition to shorter time windows. |

**Table S2:** Characteristics of case and non-cases in our study

|  | Cases | Uninfected participants |
| --- | --- | --- |
| N | 13144 | 182691 |
| Age (SD) | 14.95 (1.53) | 14.75 (1.57) |
| Male (%) | 6747 (51) | 92788 (51) |
| Dosing intervals (%) |  |  |
| 0-27 | 11663 (89) | 126038 (69) |
| 28-55 | 533 (4) | 6909 (4) |
| 56 or more | 948 (7) | 49744 (27) |

**Table S3:** Intervals of vaccination to infection between extended and regular dosing group

|  | Extended dosing group | | Regular dosing group | |
| --- | --- | --- | --- | --- |
| Days from vaccination to infection | Infection count | percentage | Infection Count | percentage |
| 0-19 | 176 | 11.9 | 6 | 0.1 |
| 20-39 | 451 | 30.5 | 12 | 0.1 |
| 40-59 | 217 | 14.7 | 18 | 0.2 |
| 60-79 | 71 | 4.8 | 17 | 0.1 |
| 80-99 | 34 | 2.3 | 21 | 0.2 |
| 100-119 | 15 | 1 | 24 | 0.2 |
| 120-139 | 30 | 2 | 43 | 0.4 |
| 140-159 | 25 | 1.7 | 200 | 1.7 |
| 160-179 | 71 | 4.8 | 1752 | 15 |
| 180-199 | 162 | 10.9 | 3191 | 27.4 |
| 200-219 | 151 | 10.2 | 3538 | 30.3 |
| 220-239 | 55 | 3.7 | 2151 | 18.4 |
| 240-259 | 14 | 0.9 | 492 | 4.2 |
| 260-279 | 7 | 0.5 | 120 | 1 |
| 280-299 | 1 | 0.1 | 65 | 0.6 |
| 300-319 | 0 | 0 | 12 | 0.1 |
| 320-339 | 1 | 0.1 | 1 | 0 |
| 0-90 | 931 | 62.9 | 66 | 0.6 |
| 91+ | 550 | 37.1 | 11597 | 99.4 |
| 0-180 | 1090 | 73.6 | 2093 | 17.9 |
| 181+ | 391 | 26.4 | 9570 | 82.1 |
